# Nutritional condition in the dry period is related to the incidence of postpartum subclinical endometritis in dairy cattle

**DOI:** 10.5713/ajas.20.0198

**Published:** 2020-06-24

**Authors:** Asako Taniguchi, Tatsuya Nishikawa, Yasuhiro Morita

**Affiliations:** 1 Okayama Prefectural Federation Agricultural Mutual Aid Association, Okayama 700-8602, Japan; 2 Graduate School of Bioagricultural Sciences, Nagoya University, Nagoya 464-8601, Japan; 3 Asian Satellite Campuses Institute, Nagoya University, Nagoya 464-8601, Japan

**Keywords:** Dairy Cattle, Dry Period, Metabolic Parameter, Nutrition, Subclinical Endometritis

## Abstract

**Objective:**

Endometritis is a major disease, that causes infertility in cattle, and is usually categorized as clinical or subclinical endometritis (SCE). The nutritional condition during the dry period is important for recovery after the last stage of the lactation period, and for postpartum production and reproduction. This study aimed to clarify the relationship between nutritional and metabolic characteristics in the dry period, and the risk of postpartum SCE.

**Methods:**

Multiparous Holstein dairy cows (n = 25, raised in a tied stall) were used. Endometrial cytological analysis was performed around 30 days post-partum, with 5% to 14% polymorphonuclear (PMN) as a cut-off point to define SCE. Serum levels of glucose, non-esterified fatty acids, β-hydroxybutyric acid (BHBA), blood urea nitrogen, total cholesterol, aspartate aminotransferase, γ-glutamyl transpeptidase, calcium, phosphorus, and magnesium were measured in the cows at the dry period to evaluate energy status, protein metabolism, and mineral metabolism.

**Results:**

The incidence of SCE in the cows was 60.0% (n = 15/25) and the mean PMN% in postpartum cows diagnosed as SCE was 8.05%±2.6%. Overall, 17 and 8 samples were collected from the cows in the far-off and close-up periods, respectively. The serum concentration of BHBA in the far-off period and serum glucose concentration in the close-up period were correlated with postpartum PMN% (r = 0.62, p<0.01; r = −0.74, p<0.05, respectively). Serum levels of calcium and magnesium in the dry period were associated with the incidence of postpartum SCE (healthy vs SCE cows, p<0.05).

**Conclusion:**

Blood levels of glucose, BHBA, calcium, and magnesium in dry periods could be useful parameters for predicting the risk of postpartum SCE. The present study also suggests that management in the close-up period is essential for promoting recovery from calving fatigue.

## INTRODUCTION

Endometritis is a disease that often causes infertility in cattle. Infection and inflammation of the uterus and cervix affects approximately one-third of dairy cows, resulting in a substantial negative impact on the probability and timing of pregnancy [1]. Disruption of the epithelium, an increase in blood flow, edema, and an influx of inflammatory cells, mostly neutrophils and lymphocytes, are often observed in the cows contracting endometritis [2,3]. Consequently, endometritis generally causes an increase in the number of artificial inseminations (AIs) required per pregnancy, a delay of the first AI timing after calving, a decrease in pregnancy rate, and an increase in calving interval.

Endometritis is defined as an inflammation of the endometrium, but not of the stratum spongiosum [3,4], and is usually categorized into the clinical (CE) or subclinical (SCE) levels. The CE typically occurs within 21 days postpartum (DPP) [5] and is defined by the detection of purulent (>50% pus) discharge between 20 and 33 DPP, or mucopurulent (approximately 50% pus and 50% mucus) discharge between 26 and 33 DPP, either by an observation of the external perineum or by use of a vaginoscope [6,7]. Conversely, the SCE is not characterized by a uterine discharge. Therefore, endometrial cytology, which is based on the presence of cellular evidence of inflammation, is currently considered to be the most useful method to diagnose both types of endometritis [3]. The percentage of polymorphonuclear (PMN) cells, calculated from the total number of the PMN and endometritis cells obtained by uterus lavage or cytobrush [8], has been usually used to define both, CE and SCE. To date, several definitions of endometritis have been postulated; 5% PMN is adequate as a diagnostic criterion for SCE at 21 to 56 DPP in some studies [9,10], while others stated that 14% PMN is a suitable threshold for diagnosing CE at 4 weeks after the delivery [11], or 18% PMN at 20 to 33 DPP is a better threshold for diagnosing CE [12]. Thus, the percentage of PMN is a particularly important indicator for improving SCE treatment.

It has been reported that a severe negative energy balance (NEB) caused by a reduced feed intake, which often causes an increase in serum non-esterified fatty acids (NEFA), and then disturbed immune function in the transition period are related to an incidence of endometritis [13,14]. However, there have been few studies on the relationship between the antepartum nutritional condition and the incidence of postpartum endometritis, particularly SCE. Most related reports to date have focused on postpartum nutrient conditions and uterine recovery and infection.

The dry period is important for recovery after the previous lactation period. The dry period can be divided into the close-up and far-off periods, which are least 3 and 4 to 6 weeks before the expected parturition, respectively. Many reports to date have indicated an importance of specific management during the close-up and far-off periods separately. In particular, the close-up period is important for fetal growth, and consequently, the cow requires more energy and protein in this period than during the far-off period [15]. Therefore, for transition success, energy supplementation should be provided to cows during the close-up period. Moreover, other studies have suggested that excessive energy intake during the far-off period had a more negative effect on metabolism after delivery than overfeeding during the close-up period [16,17]. Conversely, the far-off period is important for adaptation of the rumen environment prior to delivery [18]. Therefore, in terms of the effect of energy management, the far-off period and the close-up period should be considered separately.

The present study investigated the relationship between the antepartum nutritional condition and the incidence of postpartum SCE in dairy cattle, with a focus on the difference in metabolism conditions and energy demands among the dry, far-off, and close-up periods separately. We aimed to evaluate the risk factors related to the incidence of SCE after the transition period, and the factors essential for preventing postpartum uterine infection, by measuring the blood nutritional and metabolic parameters in the dry period.

## MATERIALS AND METHODS

### Animals and experiment design

Multiparous, healthy, Holstein dairy cows (n = 25; average age, 4.32 years) raised in Okayama Prefecture, Japan (mainly around 35°1′N 134°0′E, Tsuyama City), were selected in the present study. All cows were managed with tie-stall housing, following the recommendations of the Ministry of Agriculture and Forestry and Fishery in Japan; they were fed nutritionally adequate fodder (containing maize and whole crop rice silage [19], multiple types of hay (sudan grass, orchard grass, oats grass, timothy grass, and alfalfa), grain and soy mixed concentrate, beet pulp, and fatty and amino acid supplementation) as per the National Research Council (NRC) 2001 guidelines [20] for multiparous cows of 700 kg body weight, with approximately 35 kg (early and middle lactation) and 25 kg (middle to late lactation) milk yield per day per cow in each farm. Forage and concentration were fed separately three to five times a day depending on the farmers’ system. Water was available *ad libitum* from a water cup system. All experimental procedures were approved by the Committee for the Care and Use of Experimental Animals at Okayama Prefectural Federal Agricultural Mutual Aid Association (approval number Seiju H29–46).

We investigated the relationship between nutritional/metabolic indices and the risk of postpartum SCE in healthy transition cows in the dry period. We defined the close-up period as the last 3 weeks before parturition and the far-off period as the dry period before the close-up period. Blood sampling and body condition score (BCS) determination were performed 3 to 4 hours after the morning feeding following milking. Each cow was treated in these procedures once in the dry periods. Blood samples were collected in 10 mL plain vacuum-tubes coated with coagulation activator (Venoject, Terumo Corp., Tokyo, Japan) for serum collection, and 5 mL Ca-ethylenediaminetetraacetic acid vacuum-tubes (Venoject, Terumo Corp., Japan) for complete blood count (CBC) samples, via aseptic caudal venipuncture using an 18-gauge needle after determination of the BCS. Blood samples were immediately placed on ice before centrifugation at 1,500×g for 15 min. Serum was taken and stored at 4°C for subsequent analysis of metabolites on the same day. The mean time point of blood sampling in the close-up and far-off periods in the present study were 13.8± 5.7 days and 40.6±11.8 days (means±standard deviation), respectively.

### Cytological determination of endometritis

Cytological sampling of the endometrium was performed at approximately 30 DPP (27 to 40 DPP, median 34 DPP) by cytobrush (Metribrush, Fujihira Industry Co Ltd., Tokyo, Japan). Cytology slides were prepared by rolling the cytobrush on a clean microscope slideglass, immediately followed by fixation with a cytofixative (CytokeepII, Alfresa Pharma Co., Osaka, Japan) in the field. The slideglasses were subsequently stained with Diff-Quik (Sysmex, Kobe, Japan) for 20 seconds. The percent of polymorphonuclear leukocytes (PMN%) was assessed by counting a minimum of 200 cells at 400× magnification, to provide a quantitative assessment of endometrial inflammation. The prepared slides were assessed twice by two clinicians, one of whom collected the samples, and another did not, as in the study by Wagener et al [10]. We set 5% to 14% as the cut-off PMN% for defining the SCE; 5% PMN is considered adequate for diagnosing SCE at 21 to 62 DPP [9,10] while 14% PMN% is used as the CE threshold [11]. Cytological samples in PMN%<4, 5<PMN%<14, 15< PMN% were categorized as normal, SCE, and CE, respectively. All cows except for those diagnosed with CE were analyzed in this study.

### Measurement of serum levels of energetic and metabolic substances and complete blood counts

Serum samples were subjected to analysis for concentrations of glucose (Glu), NEFA, β-hydroxybutyric acid (BHBA), blood urea nitrogen (BUN), total cholesterol (T-chol), aspartate aminotransferase (AST), γ-glutamyl transpeptidase (GGT), calcium (Ca), phosphorus (P), and magnesium (Mg) using the automation biochemical analyzer (7180, Hitachi High-Technologies Corporation, Tokyo, Japan). Glu, NEFA, BHBA, and BCS were evaluated as parameters for the energy status, T-chol, BUN, AST, and GGT were evaluated as parameters for protein metabolism, and Ca, Mg, and P were evaluated as parameters for mineral metabolism. The CBC was evaluated using an automatic hematology analyzer (Celltak Alpha, MEK-6450, NIHON KOHDEN, Tokyo, Japan).

### Statistical analysis

The correlation coefficient between post parturition PMN% and parameters from blood analysis in the close-up and far-off periods was determined using the Pearson’s product-moment correlation test. Blood metabolic parameters were compared between non-SCE (normal, PMN%<5) cows and SCE cows using the Student’s t-test. All statistical analyses were conducted using algorithms in R version 3.5.1 (http://www.R-project.org/), stats R package.

## RESULTS

### Summary of postpartum endometrial status and blood parameters in dry periods in cows

None of the cows in this study showed placental retention or other uterine diseases after calving. The incidence of SCE in the assigned cows was 60.0% (15/25) and the mean PMN% in cows diagnosed with postpartum SCE was 8.05%±2.6% (Table 1).

### Correlation between postpartum PMN% and blood parameters in the dry period in cows

**Figure 1:**
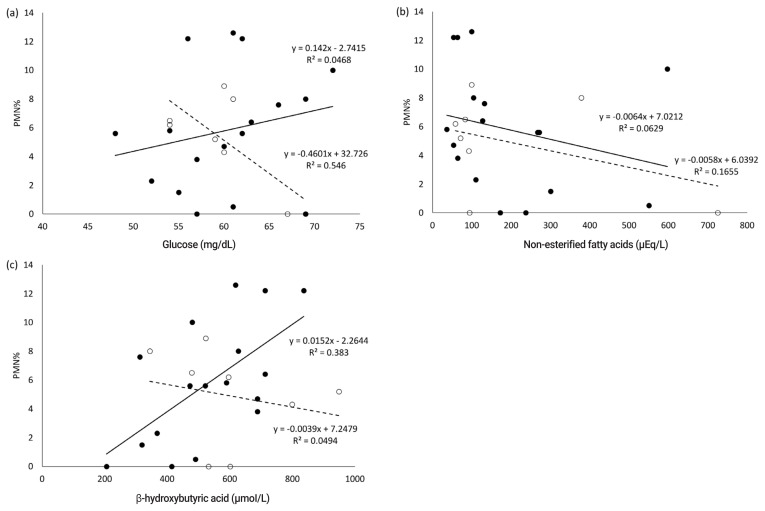
Relationship between postpartum PMN% and serum glucose (a), non-esterified fatty acids (b), and β-hydroxybutyric acid (c) concentration in the respective dry periods. Open circles show samples of the close-up period and closed circles shows samples of the far-off period.

In the present study, no abnormal CBC values were observed in the white and red blood cell count, platelet count, and hematocrit value. Blood serum parameters in the dry periods related to nutritional metabolism; the correlation coefficient between postpartum PMN% and blood serum parameters in the dry periods are shown in Table 1 and 2, respectively. The serum concentration of BHBA in the far-off period positively correlated with the postpartum PMN% (r = 0.62, p< 0.01) (Table 2; Figure 1). In the close-up period, the serum concentration of Glu had a significantly negative correlation with postpartum PMN% (r = −0.74, p<0.05), while NEFA, AST, and Ca levels were negatively correlated with PMN% (r = −0.41, −0.43, −0.51, respectively).

### Comparison of normal and subclinical endometritis cattle in terms of blood parameters in dry periods

**Figure 2:**
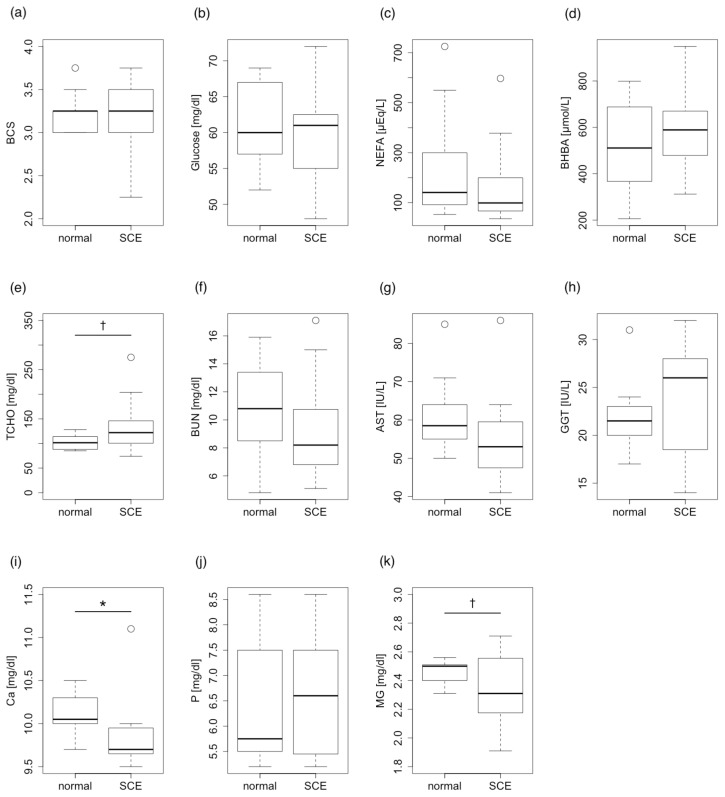
Comparison between normal cattle and subclinical endometritis (SCE) cattle in terms of postpartum blood parameters in the dry periods. (a) Body condition score (BCS), (b) Glucose, (c) non-esterified fatty acids (NEFA), (d) β-hydroxybutyric acid (BHBA), (e) total cholesterol (T-Chol), (f) blood urea nitrogen (BUN), (g) aspartate aminotransferase (AST), (h) γ-glutamyl transpeptidase (GGT), (i) calcium (Ca), and (j) phosphorus (P), and (k) magnesium (Mg). Serum concentrations of calcium and magnesium differed significantly between normal and SCE cattle (Student’s *t*-test, * p<0.05). Total cholesterol also showed a tendency for significance (Student’s *t*-test, † p<0.1).

Only mineral parameters through the dry periods were correlated with the incidence of postpartum SCE. The serum concentration of Ca and Mg differed significantly between normal (non-SCE) and SCE cattle (p<0.05). Other energy and protein metabolism parameters except for T-chol were not correlated with the incidence of SCE through the dry periods. T-chol tended to be correlated with the incidence of SCE (p<0.1) (Figure 2).

## DISCUSSION

The present study demonstrated correlations between energy parameters (Glu and BHBA) in the dry periods and PMN% after delivery, suggesting that the energy status of blood parameters in the dry periods is a risk factor for postpartum SCE, and these parameters may predict the occurrence of SCE.

In the present study, all samples were obtained from healthy cows diagnosed by physical examination; the Glu level during the close-up period was negatively correlated with PMN%. This result may indicate that the serum Glu level in the close-up period is important for postpartum uterine infections and the immune response, even if the cow appears healthy. Reynolds et al [21] reported that the splanchnic output of Glu in the transition period results almost completely from increased hepatic gluconeogenesis, and rate increases by 256%. In addition, a previous study indicated that the serum Glu level is an indicator of NEB in the close-up period and affects regulation of genes related to inflammation [14,22]. Therefore, low serum Glu concentrations before calving may indicate that the cow has an underactive liver; therefore, the serum Glu level in the close-up period may be an indicator of the liver status. Gluconeogenesis in dairy cattle is also regulated by several hormones such as insulin, glucagon, somatotropin, and cortisol, in general. Thus, the transition of blood Glu concentration for the energy status in the dry period could be affected by a cooperative response in these endocrine parameters, also finally resulting in suppression of the uterine immune function via changes in the whole-body immune response. We also described that blood levels of NEFA in the close-up period had a weak correlation with the postpartum PMN% and NEFA, that could be important parameters for improving postpartum uterine conditions. The NEFA level was reported to be a risk factor for fatty liver [23,24] and may therefore interfere with neutrophil function *in vitro* [25]. Indeed, a previous study suggested that blood levels of NEFA at a week before the expected calving time may be a useful dietary indicator for manage cows’ health during the transition period [26].

Our results indicated that BHBA in the dry periods is also essential for conserving postpartum immune responses. Other previous reports also have suggested when NEFA is over transported to the liver leading to accumulating BHBA and other ketone bodies, and when BHBA and other ketones are elevated in the dry period, immune cell function against infection is decreased after delivery [13], and increase in BHBA suppresses phagocytosis, extracellular trap formation, and removal of bacteria by PMN cells [22]. In addition, nutritional conditions during the far-off dry period significantly affects the periparturient metabolic response, regardless of the close-up feeding scheme used [17]. Evaluation of the nutritional condition by measuring blood parameters for energy status during the far-off period could be important for nutritional management during the close-up period.

There was a weak correlation between the postpartum PMN% and blood levels of AST in the close-up period in the present study, also suggesting that AST is an important parameter for improving postpartum uterine conditions. Sattler and Fürll [27] showed a significant correlation between AST and the degree of endometritis, suggesting that the energy and protein status in the transition period in dairy cows, particularly in the close-up period, is essential for improving the postpartum uterine condition and avoiding uterine infection.

The present study demonstrated that Ca levels in the close-up period showed a weak correlation with the postpartum PMN%. Previous studies showed that the blood Ca level in cows with uterine disease decreased compared to that of healthy cows; consequently, Ca is an important second messenger for PMN activation [22]. Conversely, another study showed that the demand for both, energy and Ca increases in late pregnancy period when calf growth increases [28]. Thus, serum Ca parameters in the close-up period may also by a useful indicator for predicting the postpartum uterine condition. Moreover, the present study also suggests that the plasma Mg level in the dry period was related to the SCE incidence. Mg is known to be related to Ca excretion in urine; Ca excretion tends to decrease when Mg excretion increases [29]. Therefore, a high blood Mg concentration could support maintenance of Ca concentrations in the circulation of healthy cows; this may consequently suppress the development of postpartum SCE in the dry period.

In conclusion, blood parameters of the energy status (Glu and NEFA in the close-up period, and BHBA in the far-off period) and mineral status (Ca and Mg) in the dry periods could be useful parameters for predicting the risk of onset of postpartum uterine infections. These parameters would also provide concentration management strategies for the prevention of uterine dysfunction in nutritional aspects. In addition, the present study suggests that nutritional management, particularly energy metabolism, is essential in the close-up period to promote recovery from calving fatigue in the postpartum period.

## Figures and Tables

**Table 1:** Status of postpartum endometrial infection and results of metabolic blood parameters in the dry periods

Items	Close-up (n = 8)	Far-off (n = 17)
Incidence of postpartum SCE		
n	5/8	10/17
%	60.0	
Average of postpartum PMN% in SCE cows (%)	8.05±2.6	
Body condition score	3.3±0.4	3.3±0.3
Glucose (mg/dL)	60.5±5.4	60.2±0.4
Non-esterified fatty acids (μEq/L)	200.0±236.3	190.3±166.8
β-Hydroxybutyric acid (μmol/L)	602.8±139.9	532.5±172.4
Total cholesterol (mg/dL)	99.0±8.5	131.9±50.2
Blood urea nitrogen (mg/dL)	9.5±3.5	10.1±3.7
Aspartate aminotransferase (IU/L)	60.5±11.6	56.4±10.8
γ-Glutamyl transpeptidase (IU/L)	22.0±6.3	23.2±4.7
Calcium (mg/dL)	9.9±0.3	10.0±0.4
Phosphorus (mg/dL)	6.1±1.0	6.8±1.2
Magnesium (mg/dL)	2.4±0.2	2.4±0.2

SCE, subclinical endometritis; PMN, polymorphonuclear cells.

**Table 2:** Correlation coefficient between postpartum percentage of polymorphonuclear and parameters from blood analysis

	Periods	BCS	Glu	NEFA	BHBA	T-Chol	BUN	AST	GGT	Ca	P	Mg
PMN%	Close-up (n = 8)	−0.13	−0.74*	−0.41	−0.22	0.1	−0.15	−0.43	0.2	−0.51	−0.15	0.31
	Far-off (n = 17)	0.14	0.21	−0.25	0.62**	0.25	−0.13	−0.16	0.18	−0.10	0.2	−0.11

PMN%, percentage of polymorphonuclear; BCS, body condition score; Glu, glucose; NEFA, non-esterified fatty acids, BHBA, β-hydroxybutyric acid, T-Chol, total cholesterol; BUN, blood urea nitrogen; AST, aspartate aminotransferase; GGT, γ-glutamyl transpeptidase; Ca, calcium; P, phosphorus; Mg, magnesium.

* Indicates significant differences (p<0.05) as determined by Pearson’s product-moment correlation.

** Indicates significant differences (p<0.01) as determined by Pearson’s product-moment correlation.
